# Epileptic prediction using spatiotemporal information combined with optimal features strategy on EEG

**DOI:** 10.3389/fnins.2023.1174005

**Published:** 2023-04-04

**Authors:** Lisha Zhong, Jiangzhong Wan, Fangji Yi, Shuling He, Jia Wu, Zhiwei Huang, Yi Lu, Jiazhang Yang, Zhangyong Li

**Affiliations:** ^1^School of Communication and Information Engineering, Chongqing University of Posts and Telecommunications, Chongqing, China; ^2^School of Medical Information and Engineering, Southwest Medical University, Luzhou, Sichuan, China; ^3^Research Center of Biomedical Engineering, Chongqing University of Posts and Telecommunications, Chongqing, China; ^4^Central Nervous System Drug Key Laboratory of Sichuan Province, Luzhou, Sichuan, China; ^5^Yongchuan Women and Children Hospital, Chongqing, China

**Keywords:** epilepsy, spatiotemporal features, fuzzy entropy, power spectral density, brain network, EEG

## Abstract

**Objective:**

Epilepsy is the second most common brain neurological disease after stroke, which has the characteristics of sudden and recurrence. Seizure prediction is seriously important for improving the quality of patients’ lives.

**Methods:**

From the perspective of multiple dimensions including time-frequency, entropy and brain network, this paper proposed a novel approach by constructing the optimal spatiotemporal feature set to predict seizures. Based on strong independence and large information capabilities, the two-dimensional feature screening algorithm is performed to eliminate unnecessary redundant features. In order to verify the effectiveness of the optimal feature set, support vector machine (SVM) was used to classify the preictal and interictal states on both the Kaggle intracranial EEG and CHB-MIT scalp EEG dataset.

**Results:**

This model achieved an average accuracy of 98.01%, AUC of 0.96, F-Score of 98.3% and FPR of 0.0383/h on the Kaggle dataset; On the CHB-MIT dataset, the average accuracy, AUC, F-score and FPR were 95.93%, 0.92, 94.97% and 0.0473/h, respectively. Further ablation experiments have confirmed that the temporal and spatial features fusion has better performance than the individual temporal or spatial features.

**Conclusion:**

Compared to the state-of-the-art methods, our approach outperforms most of these existing techniques. The results show that our approach can effectively extract the spatiotemporal information of epileptic EEG signals to predict epileptic seizures with high performance.

## 1. Introduction

Epilepsy is a neurological disease of brain activity, caused by excessive and synchronous electrical discharges. As the second most common disease after stroke, epilepsy affects approximately 70 million people worldwide, which is nearly 1% of the global population, and approximately 80% in developing countries according to the World Health Organization. The sudden and recurring seizures are catastrophic for patients, easily resulting in loss of consciousness, injury and even death by accidents (Lenkov et al., 2013; Zhong et al., 2022c). Therefore, reliable seizure prediction is prime important, as it can greatly improve the quality of patients lives. Electroencephalogram (EEG) which reflects the discharges of neurons, provides plenty of valuable information about brain activities. Due to the advantages of cheap price and high temporal resolution, EEG becomes one of the most useful tools in the diagnosis and prediction of epilepsy (Freestone et al., 2017; Jia et al., 2022; Peng et al., 2022). Contrasting to the obvious difference in the ictal states, EEG signals in the preictal states are similar to the interictal states, which leads to a great challenge in how to accurately forecast epileptic seizures. Therefore, the essence of epilepsy prediction is to identify preictal EEG signals, that is, to accurately distinguish between preictal and interictal states (Chu et al., 2017).

Over the past few decades, with the development of machine learning and deep learning, seizure prediction based on EEG recordings has attracted extensive attention. Nejedly et al. (2019) proposed an automatic seizure prediction approach using CNN with an average sensitivity of only 79%. Usman et al. (2021) extracted handcrafted and automatic features, which were then fed into an ensemble classifier of SVM, CNN, and LSTM, and finally achieved a high accuracy of 95.5%. Chen et al. (2021) put forward an online seizure prediction method with an average sensitivity of 84%. Successive variational mode decomposition and transformers deep learning network has been proposed and achieved an average sensitivity of 0.86 and FPR of 0.18/h on iEEG signals (Wu et al., 2022). Although the good performance of deep learning approach in seizure prediction, its lack of interpretability has limited its clinical application. Therefore, this paper still focuses on machine learning that requires handcrafted features. The prediction performance using machine learning mainly depends on whether the EEG features are effectively extracted and screened.

Entropy, as a good non-linear feature for the complexity evaluation of EEG signals, has been widely proposed for seizure prediction in previous studies (Xiang et al., 2015; Song and Zhang, 2016; Zhang et al., 2018). Zhang et al. (2018) put forward the fuzzy distribution entropy to automatically detect seizure. Sample entropy-based features and extreme learning machine to distinguish interictal and preictal iEEG signals with a sensitivity of 86.75% and a specificity of 83.80% (Song and Zhang, 2016). Some methods are focused on time-frequency features such as power spectral density (PSD) (Zhong et al., 2022b), empirical mode decomposition (EMD) (Cho et al., 2016), and wavelet transform (Faust et al., 2015; Sharma et al., 2015). A dual self-attention residual network proposed by Yang et al. (2021) has extracted the spectrograms by using a short-time Fourier transform and achieved an accuracy of 92.07% on 13 patients in the CHB-MIT dataset. Another important feature during the process of epileptic seizures is synchronization, which can quantify the degree of mutual coupling among brain regions. Previous studies have reported that EEG synchronization can be employed to predict seizures (Ibrahim and Majzoub, 2017; Zhang et al., 2021). Some researchers combined the spatiotemporal features to construct the multi-dimensional feature set. Zhong et al. (2022a) proposed a novel method based on both entropy and synchronization of iEEG signals, and achieved an accuracy of 82.95% on the Kaggle dataset. However, the frequency domain has not been considered.

Although spatial synchronization, entropy, or time-frequency features could be utilized to predict seizures, most of these methods only consider a certain aspect of EEG signals characteristics. Even some methods with multiple features have been resulting in unsatisfactory performance due to not implementing proper screening algorithms. Most of those current methods can only achieve good results in a specific dataset. On the one hand, the reason is that EEG signals in different datasets lack unified labels. On the other hand, the types of epilepsy are diverse, and the dynamics of epilepsy vary greatly among different patients. Therefore, the typical EEG features of some patients may not be suitable for others. Seizures can be seen as the accumulation of abnormal fluctuations over time, and then spread across brain regions through spatial synchronicity, and are also affected by waveforms in different frequency bands. To solve these problems mentioned above, this paper extracts comprehensive multi-dimensional features including non-linearity, time-frequency and spatial domains from the perspective of spatiotemporal information. In order to select the optimal feature set, a feature screening algorithm that takes into account independence and information capabilities is designed. And then the optimal feature set was as the input to the SVM for training and testing. Our approach achieved good prediction performances on both scalp and intracranial EEG signals.

## 2. Materials and methods

### 2.1. Dataset description

In this study, the proposed model is tested on two public EEG datasets, the CHB-MIT scalp EEG dataset^1^ and the Kaggle competition iEEG dataset.^2^ These two public EEG datasets included long-term EEG signals and multiple seizures have been recorded for each subject.

CHB-MIT dataset consists of continuous scalp EEG recordings of 23 epileptic patients from Boston Children’s Hospital over many days. Multi-channel EEG signals were recorded with a sampling rate of 256 Hz using the international 10−20 system. In this paper, the preictal state was defined as a 30 min signal before the seizure onset; and the interictal state was determined as at least 4 h far away from any seizure. The upcoming seizure is excluded with an interval of less than half an hour between two adjacent seizures to ensure the preictal states with the length of 30 min. Patients with at least three recorded preictal and interictal states were screened. The reason is that less than three preictal or interictal states would lead to an overfitting problem. A total of 14 patients are available for considering all these definitions and constraints. Table 1 summarizes the details of these 14 patients used in our experiments.

**TABLE 1:** Summary of the 14 patients in the CHB-MIT dataset.

Patients	Gender	Age	No. of seizures	Total record time (h)	No. of electrodes
chb01	Female	11	7	40.55	23
chb03	Female	14	7	28	23
chb05	Female	7	5	39	23
chb06	Female	1.5	9	66.7	23
chb07	Female	14.5	3	68.1	23
chb08	Male	3.5	5	20	23
chb09	Female	10	4	67.8	23
chb10	Male	3	7	50	23
chb14	Female	9	8	26	23
chb15	Male	16	20	40	31
chb18	Female	18	6	36	22
chb20	Female	6	8	29	28
chb21	Female	13	4	33	28
chb23	Female	6	7	28	28

The Kaggle competition dataset consists of intracranial EEG signals recorded from five dogs with naturally occurring epilepsy using an ambulatory monitoring system. The iEEG recordings were collected from 16 electrodes with a sampling rate of 400 Hz. Preictal states were determined as 1 h before seizure onset and 1 h interictal iEEG signals were restricted to be at least 1 week before or after seizure in this dataset. All canines had experienced at least four seizures, and a total of 44 seizures were recorded in this experiment. The detailed information is shown in Table 2.

**TABLE 2:** Information for the canines in the Kaggle competition dataset.

Dogs	No. of seizures	Interictal states (10 min)	Preictal states (10 min)	No. of electrodes
Dog1	4	480	24	16
Dog2	7	500	42	16
Dog3	12	1440	72	16
Dog4	16	804	97	16
Dog5	5	450	30	15

### 2.2. Methodology

**FIGURE 1:**
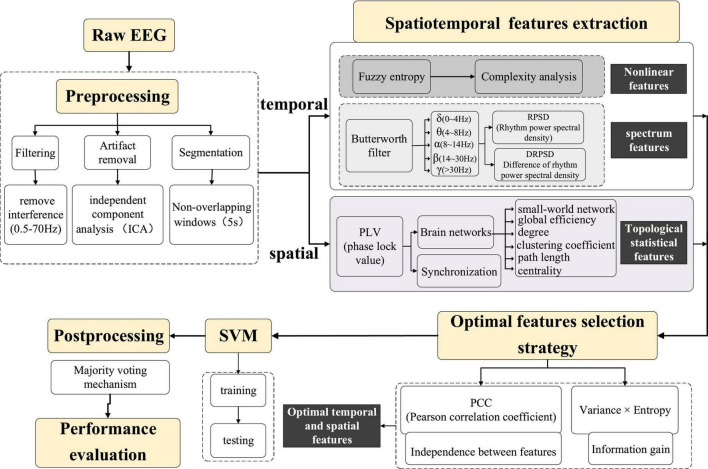
The flow chart of the algorithm.

The flow chart of the algorithm for seizure prediction using the spatiotemporal information with the optimal features strategy is shown in Figure 1, and the detailed steps are as below:

#### 2.2.1. Pre-processing

**FIGURE 2:**
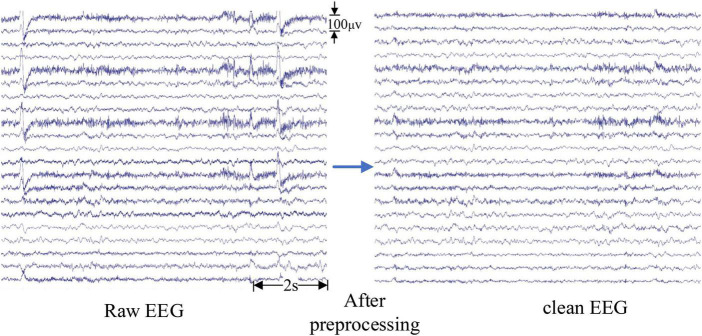
The clean electroencephalogram (EEG) signals after pre-processing.

The amplitude of scalp EEG signals is weak, making it easily disturbed by the external environment, such as electrode contact, power frequency interference, etc. In addition, various physiological activities inside the human body also produce artifacts, such as electrooculogram (EOG) artifacts caused by eye movement and blinking, electromyogram (EMG) artifacts caused by muscle shaking, and electrocardiogram (ECG) artifacts caused by heart beating. These artifacts often affect and interfere with the experimental results. In comparison, intracranial EEG signals are less susceptible to interference, and their signals are relatively clean and less affected by the environment. Therefore, different pre-processing procedures are applied to intracranial and scalp EEG signals, as described below:

The pre-processing of intracranial EEG signals is relatively simple to avoid removing valuable information. Baseline drift was removed by subtracting the mean value of the iEEG signal from each data point (Wu et al., 2009). Then a simple fourth-order Butterworth bandpass filter with a range of 0.5∼70 Hz was used to filter the iEEG signal. For scalp EEG, in addition to the above-mentioned pre-processing steps, the following measures were taken to remove interference: a 50 Hz notch filter was used to remove the power-line interference and independent component analysis (ICA) has been developed to effectively remove artifacts in EEG signals (Du et al., 2016). Artifacts such as eye movement, eye blink, and muscle artifacts were removed by using ICA in the EEGLAB toolbox (Delorme and Makeig, 2004) with the guidelines (Urigüen and Garcia-Zapirain, 2015). Artifacts that cannot be removed through signal processing, such as severe crying or intense head movement, are excluded directly from the experimental data. The results after pre-processing are shown in Figure 2. The EEG signals become smoother, and the burr, interference as well as EOG artifacts are effectively removed from the raw EEG signals.

The long-term continuous EEG recordings need to be segmented. The duration of the segment is commonly performed from 5 to 30 s. In our method, a 5 s non-overlapping moving window was used to divide the clean EEG signals into 5 s epochs. EEG analysis and feature extraction were performed with the software package MATLAB R2016b (The MathWorks, Inc., Natick, MA, United States) and its EEGlab and statistics toolbox.

#### 2.2.2. Features extraction

Accurate extraction of the EEG features that can distinguish between preictal and interictal states is the key to improving the prediction accuracy. This paper analyses epileptic EEG signals from multiple dimensions such as non-linear, time-frequency and brain networks with the purpose of deeply mining the signals’ spatiotemporal features. The temporal features include the non-linear feature fuzzy entropy and the spectral features; and the spatial features are jointly constructed from the statistical parameters and the synchronization of the brain network. The principle of these spatiotemporal features is as follows:

##### 2.2.2.1. Fuzzy entropy

Entropy originally measures the degree of chaos in a thermodynamic system, and it can also describe the probability of the occurrence of new events in a time-series signal. Fuzzy entropy (FuzzyEn) was proposed by Chen et al. (2007) to measure the complexity of time series, which is then used as a non-linear feature to evaluate the complexity of EEG. FuzzyEn can be obtained by the following steps:

For a time series of *N* points *U* = {*u*(*i*),*i* = 1,…,*N*}, *m*-dimensional vectors *X*(*i*) are formed as:


(1) $$X\,\left(i\right)=\left[u\,\left(i\right),u\,\left(i+1\right),\cdots ,u\,\left(i+m-1\right)\right]-u_{0}\,\left(i\right),$$

i=1,2,⋯,N-m+1

where $u_{0}\,\left(i\right)=\frac{1}{m}\,\sum_{j=0}^{m-1}u\,\left(i\,j\right)$ and *m* indicates the embedding dimension.

The distance matrix $d_{ij}^{m}$ between vectors *X*(*i*) and *X*(*j*) is constructed as:


$$d_{i\,j}^{m}=d\left[X\left(i\right),X\left(j\right)\right]=\underset{p=1,2,\cdots ,m}{m\,a\,x}\{|u\left(i+p-1\right)$$

(2) $$\;-u_{0}\left(i\right)|-|u\left(j+p-1\right)-u_{0}\left(j\right)|\}$$

where *k* indicates the sequence number of elements of the reconstructed vector.

The similarity degree $A_{ij}^{m}$ can be calculated through the fuzzy function *A(x)*:


(3) $$A\,\left(x\right)=\left\{ \begin{array}{cc} 1, & x=0\\ \exp\left[-l\,n\,\left(2\right)\,{\left(\frac{x}{y}\right)}^{2}\right], & x>0 \end{array}\right.$$

(4) $$A_{i\,j}^{m}=e\,x\,p\,\left[-l\,n\,\left(2\right)\cdot {\left(d_{i\,j}^{m}/r\right)}^{2}\right],j=1,2,N-m+1$$

a⁢n⁢d⁢j≠i

Define the function Φ*^m^*(*r*) as:


(5) $$C_{i}^{m}\,\left(r\right)=\frac{1}{N-m}\,\sum_{j=1,j\neq i}^{N-m+1}A_{i\,j}^{m}$$

(6) $$\Phi^{m}\,\left(r\right)=\frac{1}{N-m+1}\,\sum_{i=1}^{N-m+1}C_{i}^{m}\,\left(r\right)$$

Similarly, Φ^*m* + 1^(*r*)can be calculated by the above process. For a finite set, FuzzyEn can be estimated by


(7) $$F\,u\,z\,z\,y\,E\,n\,\left(m,r,N\right)=l\,n\,\phi^{m}\,\left(r\right)-l\,n\,\phi^{m+1}\,\left(r\right)$$

This paper set the dimension *m* = 2 and the tolerance*r* = 0.2×*std*(*standarddeviation*).

##### 2.2.2.2. Power spectral density (PSD)

Welch method was used to calculate the PSD of EEG signals with the advantages of fast calculation speed and multiple windows for selection (Zhang and Parhi, 2016). In accordance with Welch’s periodogram method, the PSD of the EEG segment in each frequency band was estimated by the following steps (Welch, 1967):

First, the EEG signal *x*_*N*_(*n*),*n* = {0,1,,*N*−1} is divided into *L* segments. Each segment has *M* points and the PSD for the *i*th segment is obtained as:


(8) $$P_{i}\,\left(w\right)=\frac{1}{U}\,{\left|\sum_{n=0}^{M-1}x_{N_{i}}\,\left(n\right)\,d_{2}\,\left(n\right)\,e^{-j\,w\,n}\right|}^{2},i=1,2,M-1$$

where $U=\frac{1}{M}\,\sum_{n=0}^{M-1}d_{2}^{2}\,\left(n\right)$ and *d*_2_(*n*) is the window function.

Then, the PSD of the *x*_*N*_(*n*) can be expressed as:


(9) $$P\,\left(w\right)=\frac{1}{L}\,\sum_{i=1}^{L}P_{i}\,\left(w\right)$$

Mathematically, the PSD in the *i*th frequency band (*delta, theta, alpha, beta, and gamma*) is calculated as (Zhang and Parhi, 2016):


(10) $$P_{i}=\log\sum_{\omega \in \mathrm{band}\,i}P\left(\omega \right),\left\{i=delta,theta,alpha,beta,gamma.\right\}$$

where delta (0∼4 Hz), theta(4∼8 Hz), alpha(8∼14 Hz), beta(14∼30 Hz) and gamma (>30 Hz). Therefore, Rhythm Power Spectral Density (RPSD) can be calculated according to formula (10). Spectral power ratio (SPR) represents the difference between the PSDs in two different bands in the same time window. SPR of the spectral power in band *k* over that in band *l* can be computed as:


(11) $$P_{k-l}=P_{k}-P_{l}$$

where *P*_*k*_ represents the PSD in band *k*; *P*_*l*_ represents the PSD in the band *l*.

For a single-channel EEG signal, all possible combinations of five frequency bands lead to a total number of 10 SPR and 5 RPSD features. SPR and RPSD features have been confirmed to be good features for seizure detection (Bandarabadi et al., 2014) and prediction (Parhi and Zhang, 2013). Compared to the RPSD features, certain SPR features are stronger indicators of an upcoming seizure (Zhang and Parhi, 2016).

##### 2.2.2.3. Spatial features based on brain networks

PLV, as an independent of amplitude, is suitable to measure the phase synchronization of EEG signals, which can be computed as follow (Lachaux et al., 1999):


(12) $$P\,L\,V\,\left(t,f\right)=\frac{1}{N}\,\left|\sum_{n=1}^{N}\exp\left(j\,\left\{\Delta \,\phi \,\left(t,f\right)\right\}\right)\right|$$

where ΔΦ(*t*,*f*) is the instantaneous phase difference between a pair of EEG channels at time *t* and frequency *f*. Taking channels 1 and 2 for example, ΔΦ(*t*,*f*) is calculated as:


(13) $$\Delta \,\Phi \,\left(t,f\right)=\Phi_{c\,h\,1}\,\left(t,f\right)-\Phi_{c\,h\,2}\,\left(t,f\right)$$

where Φ_*ch*1_(*t*,*f*) and Φ_*ch*2_(*t*,*f*) are the instantaneous phases of the EEG signals in channel 1 and channel 2, respectively. Instantaneous phase ϕ(*t*) is obtained by the Hilbert transform (Ihlen, 2009). The value of PLV ranges from 0 to 1. The larger the PLV value, the stronger the synchronization of the signal, and vice versa.

From the perspective of graph theory, the complex phase-synchronized brain network established by PLV belongs to the undirected connection graph, which contains rich topological statistical features. In addition to the synchronization, 6 statistical features including small-world attributes (Humphries et al., 2006), global efficiency, degree, clustering coefficient, characteristics path length and eigenvector centrality (Rubinov and Sporns, 2010) have been chosen as the spatial features.

#### 2.2.3. Optimal spatiotemporal feature set selection

In summary, for each electrode, 23 spatiotemporal features, which include 1 FuzzyEn, 5 RPSD, 10 SPRs, and 7 topological statistical features are extracted every 5 s. As more and more features have been extracted for multi-channel long-term EEG signals, there are a large number of irrelevant redundant features in the spatiotemporal features. This greatly reduces the performance of the classifier, causing the curse of dimensionality. Therefore, the feature selection algorithm is essential. The critical strategy is to select the most important EEG features that can best express the characteristics of preictal states, thereby removing the redundant features to reduce the dimension of features. This paper proposed a two-dimensional feature selection algorithm based on independence and information capability in order to form the optimal epilepsy spatiotemporal feature set. The stronger the independence of the features (lower the correlation between the features) contained in the feature set, the less redundancy of the features is guaranteed. The larger the information content of the feature set, the more comprehensive and effective features can be obtained to measure the epileptic EEG signals. Therefore, the spatiotemporal feature set selection algorithm should satisfy strong independence and large information.

Pearson correlation coefficient (Barakchian et al., 2020) has been used to calculate the correlation between features. The independence of the *i*th feature *ind*_*i*_can be evaluated as:


(14) $$r_{i,k}=\frac{\sum_{j=1}^{n}\left(f_{j\,i}-\bar{f_{i}}\right)\,\left(f_{j\,k}-\bar{f_{k}}\right)}{\sqrt{\sum_{j=1}^{n}{\left(f_{j\,i}-\bar{f_{i}}\right)}^{2}\,{\left(f_{j\,k}-\bar{f_{k}}\right)}^{2}}}$$

i,k=1,2,⋯,m(i≠k);j=1,2,⋯,n.

(15) $$i\,n\,d_{i}=\sum_{k=1}^{m}\left(1-\left|r_{i,k}\right|\right)$$

where *r*_*i,k*_ represent Pearson correlation coefficient between two features *i* and *k* with *n* samples; *f*_*ji*_ and *f*_*jk*_ represent the value of the features *i* and *k* at the *j*th sample, $\bar{f_{i}}$ and $\bar{f_{k}}$ represent the means of *f*_*ji*_ and *f*_*jk*_, respectively. *m* is the number of all features and *n* is the total number of samples.

In terms of the features’ information capacity, this paper used the variance entropy product to measure the amount of information (Daoud and Bayoumi, 2019). Variance is used to evaluate the fluctuation of features, and entropy can measure the complexity of features. First, calculate the variance and information entropy of the *i*th feature. The variance σ^2^(*X*_*i*_) and the entropy *H*(*X*_*i*_) were defined by formulas (17) and (18), respectively. Then, multiply the variance and entropy to measure the information of features (formula 19). Finally, the features are selected with the highest variance entropy product.


(16) $$\sigma^{2}\,\left(X_{i}\right)=\frac{1}{N}\,\sum_{j=1}^{N}{\left(x_{i}\,\left(j\right)-\mu_{i}\right)}^{2}$$

(17) $$\left(X_{i}\right)=-\sum_{j=1}^{N}p\,\left(x_{i}\,\left(j\right)\right)\,\log_{2}p\,\left(x_{i}\,\left(j\right)\right)$$

(18) $$i\,n\,f_{i}=ó\,\left(X_{i}\right)\cdot H\,\left(X_{i}\right)$$

where *X_i_*, μ_*i*_ and *N* are the *i*th feature, the mean of the *i*th feature, and the total number of features, respectively. *p*(*x*_*i*_(*j*)) is the probability mass function of the *i*th feature.

In the two-dimensional space with independence as the abscissa and information amount as the ordinate, the features with high independence and a large amount of information are screened out. *Score*_*i*_ is defined to represent the independence and information of the *i*th feature as:


(19) $$S\,c\,o\,r\,e_{i}=i\,n\,f_{i}\cdot i\,n\,d_{i}$$

Then features with high *Scores* are selected to form the optimal spatiotemporal feature set.

#### 2.2.4. Classification and post-processing

**FIGURE 3:**
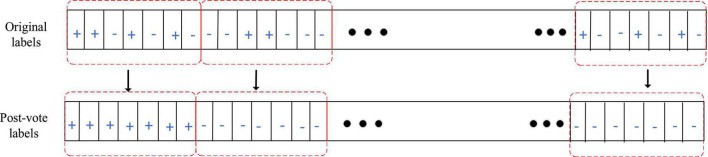
Majority voting diagram.

SVM, as a common classifier in EEG signals, is used for training and classification. The kernel function selected for SVM in this paper is the default parameter radial basis function: *K*(*x*,*x*_*i*_) = *exp*(−ã|*x*−*x*_*i*_|2). In order to improve the recognition performance of the algorithm, post-processing is to reprocess the classification results of EEG signals in continuous time windows. Specifically, a majority vote is performed on the output results within a 1 min time window and the majority voting diagram is shown in Figure 3.

### 2.3. Evaluation metrics

In order to verify whether the epileptic EEG spatiotemporal feature set constructed by the algorithm proposed in this paper can distinguish between the preictal and interictal states, four indicators including accuracy rate (ACC), the area under the receiver operating characteristic curve (AUC), F-score, and false positive rate (FPR) were introduced to evaluate the performance. The evaluation measures are defined as follows (Moridani et al., 2019):


(20) $$A\,C\,C=\frac{T\,N+T\,P}{T\,N+F\,P+T\,P+F\,N}\times 100\%$$

(21) $$F-s\,c\,o\,r\,e=\frac{2\,T\,P}{2\,T\,P+F\,P+F\,N}\times 100\%$$

(22) $$F\,P\,R=\frac{F\,P}{F\,P+T\,N}\times 100\%$$

where *TP*, *TN*, *FP*, and *FN* refer to true positive, true negative, false positive and false negative, respectively.

## 3. Results and discussion

### 3.1. Spatiotemporal feature analysis

#### 3.1.1. Fuzzy entropy

**FIGURE 4:**
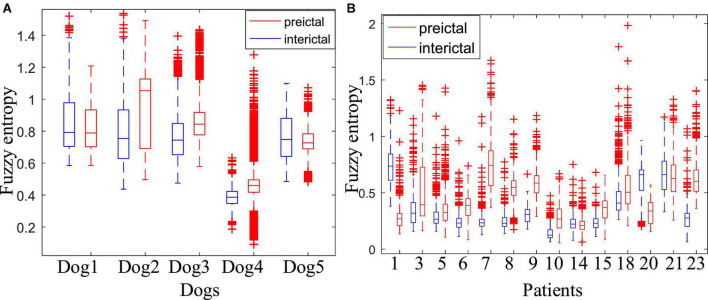
Comparison of the fuzzy entropy between the preictal and interictal states. **(A)** Is the fuzzy entropy for dogs in the Kaggle dataset and **(B)** is for patients in the CHB-MIT dataset.

Figure 4 shows the comparison of the fuzzy entropy between the preictal states and the interictal states. The change of fuzzy entropy for each sample is inconsistent. For Dog2, Dog3, and Dog4, the fuzzy entropy in the preictal state was significantly greater than that of the interictal state, making it relatively easy to distinguish between the preictal and the interictal states. However, fuzzy entropy of Dog1 and Dog5 are basically overlapped in these two states, making it difficult to distinguish between these two states. For epileptic patients in the CHB-MIT dataset, except patients chb01, chb14, and chb20, the fuzzy entropy in the preictal states is greater than that of the interictal states. The experimental results show that the fuzzy entropy of the epileptic EEG in most of the patients is significantly larger than the interictal states, indicating that the brain activity has changed before the seizure. And the higher complexity of EEG occurs in the preictal states, indicating the upcoming seizure. The experimental results also show that the fuzzy entropy of epileptic EEG signals has large individual differences. Some samples have good classification effect, while others are not sensitive, making it not suitable for each subject. Therefore, although fuzzy entropy can be regarded as an important feature to predict epileptic seizures, it is not suitable as a single feature of epileptic EEG for seizure prediction.

#### 3.1.2. PSD

**FIGURE 5:**
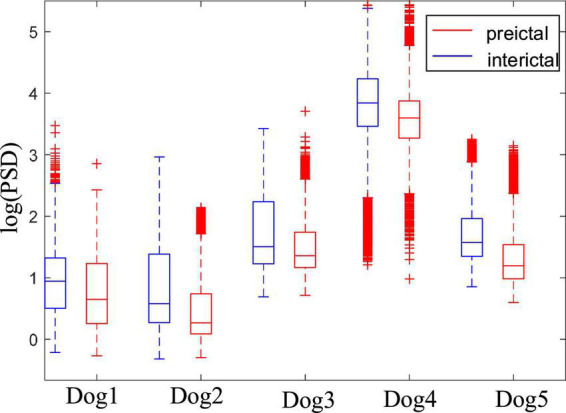
Comparison of power spectral density (PSD) between preictal and interictal states for five canines in the Kaggle dataset.

**FIGURE 6:**
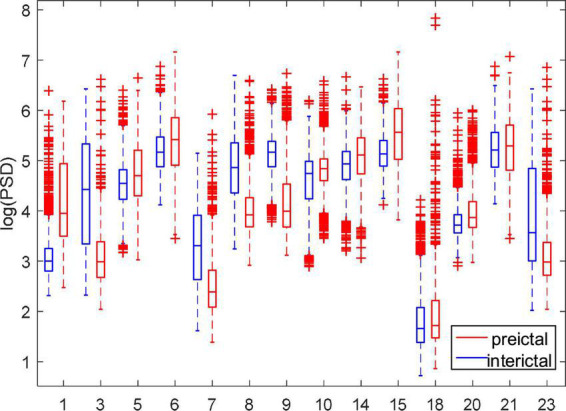
Comparison of PSD between preictal and interictal states for 14 patients in the CHB-MIT dataset.

Figure 5 shows the comparison of PSD between the preictal and interictal iEEG signals for five canines. The results indicate that the PSDs in the interictal states were higher than that in the preictal states. And there are more outliers in both the preictal and interictal states for Dog4. The individual differences exist in the PSD of epileptic EEG signals. As shown from Figure 5, there is some overlap in the overall trend between the interictal states and the preictal states for each subject. Figure 6 compares the PSD of preictal and interictal scalp EEG of 14 patients in the CHB-MIT database. Most of these patients had significant differences in PSD between the preictal and interictal states. Especially for patients chb01, chb03, chb07, chb08, and chb09, the PSD of their EEG signals could be available for distinguishing between preictal and interictal states. However, for an individual patient (such as patient chb21), it is almost impossible to classify the preictal and interictal states using PSD due to the overlapping of PSD in these two states. Therefore, only using PSD as a feature to classify the interictal and the preictal EEG signals is inappropriate and cannot achieve good prediction accuracy.

**FIGURE 7:**
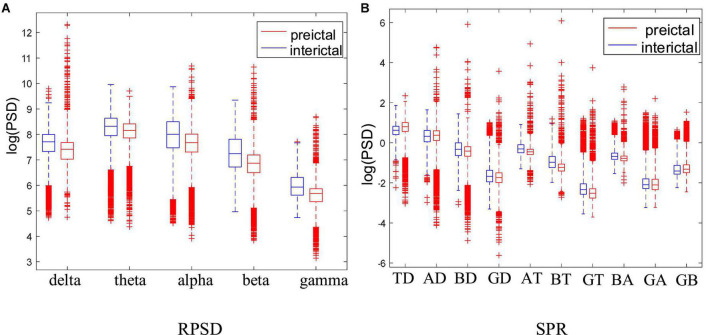
Comparison of the RPSD and DRPSD in Dog4. **(A)** Is the RPSD and **(B)** is the SPR. Note that the abscissa label of panel **(B)** represents the difference values obtained by subtracting two different RPSDs. Specifically, TD, theta minus delta; AD, alpha minus delta; BD, beta minus delta; GD, gamma minus delta; AT, alpha minus theta; BT, beta minus theta; GT, gamma minus theta; BA, beta minus alpha; GA, gamma minus alpha; GB, gamma minus beta.

In order to further analyze the PSD variation of each rhythm, this paper calculated the RPSD and SPR of each subject. Taking Dog4 as an example, the results are shown in Figure 7. Except for theta rhythm, RPSD is higher in the interictal states than in the preictal states. RPSD of beta and gamma rhythms have more outliers in the preictal states. Meanwhile, the RPSD of theta has no significant differences between the preictal and the interictal states while the SPR associated with theta rhythm was significantly different. For BT (beta-theta) and AT (alpha-theta), the SPTs in the interictal states are greater than those in the preictal states, and more outliers appear in preictal states. These results suggest that BT and AT may be good features to distinguish between interictal and preictal states. However, some other SPRs, such as GA (gamma-alpha) and GB (gamma-beta), are difficult to distinguish these two states. For each subject, some specific features may be more suitable. Therefore, RPSD and SPR are combined to further screen the optimal features set.

#### 3.1.3. Spatial features based on brain networks

**FIGURE 8:**
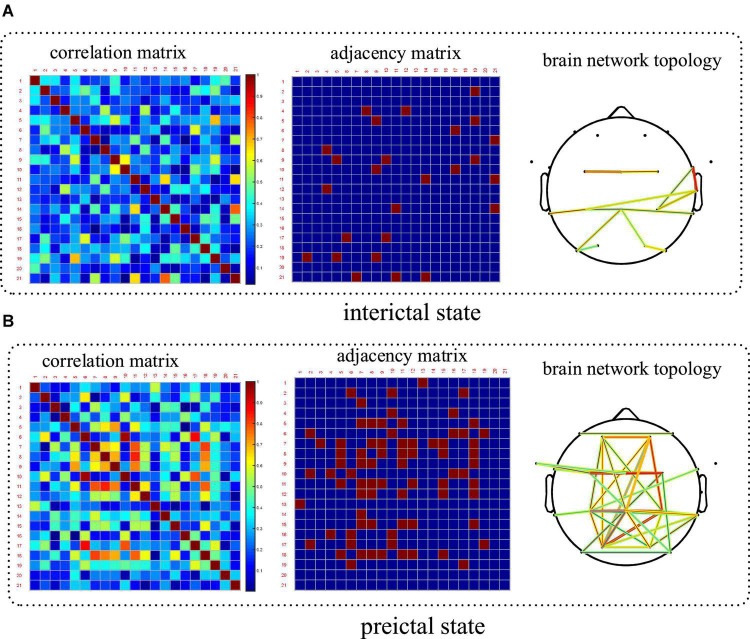
Comparison of the spatial features based on brain network between the interictal and preictal states. Patient number chb06 has been taking as an example. **(A)** Brain network in the interictal state and **(B)** is in the preictal state.

PLV which is a good measure of phase synchronization was used to construct brain networks in this paper. CHB-MIT scalp EEG dataset has electrode position information, which makes the display of the constructed brain network more convenient and intuitive. Therefore, taking a patient in this dataset as an example to compare the spatial features between the preictal and interictal states, and the results are shown in Figure 8. The results showed that the synchronization between the electrodes in the preictal states was higher than that in the interictal states, indicating that the abnormal EEG signals had begun to spread and affect more brain regions before the seizure onset. Spatial coupling and connectivity can also be observed from the brain network topology drawn from the adjacency matrix. Topological connectivity has been altered in the preictal states with significant enhancement and been covered with most areas of the brain. The results show that the statistical characteristics of network topology can effectively extract epilepsy information before seizures and can be further applied to seizure prediction.

### 3.2. Comparing the optimal spatiotemporal features selection algorithms

An ablation study is carried out to verify the feature selection algorithm on both the Kaggle competition and CHB-MIT datasets. Four feature selection schemes, namely without features selection, independence-based, information-based, and the feature score proposed in this paper, are compared. The results are shown in Table 3, the accuracy rate without feature selection is the lowest, followed by independent-based or information-based feature selection with an accuracy rate below 80%. The feature score algorithm composed of the independence and the information achieved the highest accuracy, reaching 98.01% on the Kaggle competition dataset and 95.93% on the CHB-MIT dataset. The experimental results illustrate that our proposed feature selection algorithm can effectively extract the optimal spatiotemporal features and accurately distinguish between the preictal and interictal states.

**TABLE 3:** Comparison of four feature selection schemes.

Dataset	ACC (%)			
	Without features selection	Independence-based	Information-based	Feature score
Kaggle competition	69.86 ± 27.19	78.07 ± 8.27	75.84 ± 4.06	98.01 ± 2.60
CHB-MIT	66.14 ± 18.11	73.57 ± 3.57	79.96 ± 2.75	95.93 ± 5.74

### 3.3. Prediction performance verification

#### 3.3.1. Performance evaluation of seizure prediction based on the Kaggle dataset

Table 4 shows the performances of preictal and interictal iEEG signal classification for five epileptic dogs on the Kaggle dataset. Our method achieved an average ACC of up to 98.01%, an average AUC of 0.96 and an average F-score is 98.30% with an FPR of only 3.83%. Dog4 has the highest prediction accuracy, reaching 100%. The reason may be that, on the one hand, our proposed spatiotemporal features can better express the epileptic information about impending seizures for Dog4; on the other hand, Dog4 has the largest amount of preictal iEEG data (16 seizures), making more data available for training and testing, which improves the performance of the classifier. Compared to other canines, the accuracy of Dog5 is relatively low, especially the FPR is up to 11%. The reason may be the small sample size (only 5 seizures occurred). Alternatively, it is also possible that Dog5 has a damaged electrode that has been removed in the pre-processing, which may lead to a loss of spatial information. Overall, the optimal spatiotemporal features proposed in this paper can well distinguish the preictal and interictal states.

**TABLE 4:** Performance evaluation of our proposed method on the Kaggle dataset.

Subjects	ACC (%)	AUC	F-score (%)	FPR (%)
Dog1	99.65 ± 0.69	0.99 ± 0.01	99.66 ± 0.69	0.70 ± 1.39
Dog2	96.28 ± 9.68	0.93 ± 0.19	97.03 ± 7.70	7.43 ± 19.35
Dog3	99.77 ± 0.60	0.99 ± 0.01	99.76 ± 0.61	0.13 ± 0.44
Dog4	100.00 ± 0.00	1.00 ± 0.00	100 ± 0.00	0.00 ± 0.00
Dog5	94.34 ± 7.93	0.89 ± 0.16	95.04 ± 6.77	11.04 ± 15.94
Total	98.01 ± 2.60	0.96 ± 0.05	98.30 ± 2.19	3.83 ± 5.05

To explore the impact of features on epilepsy prediction, an ablation study is conduct to compare our proposed method with two other methods that use either temporal features or spatial features alone. As shown in Table 5, only temporal features are used as the input to the classifier with average accuracy, AUC, F-score, and FPR of 87.7, 0.84, 87.91, and 4.42%, respectively. Using only brain network-based spatial features to distinguish between interictal and preictal states, the results yielded an average accuracy of 81.75%, an average AUC of 0.73, an F-score of 80.91%, and an FPR of 19.64%. It is evident that our proposed method which fuses spatial and temporal features greatly improves the performance of seizure prediction. Therefore, it is necessary to consider both spatial and temporal features when analyzing epileptic EEG signals.

**TABLE 5:** Comparing the proposed spatiotemporal features method for seizure prediction to the temporal or spatial features method on the Kaggle dataset.

Features		ACC (%)	AUC	F-score (%)	FPR (%)
Spatial features	Brain networks	81.75 ± 8.72	0.73 ± 0.12	80.91 ± 10.89	19.64 ± 15.01
Temporal features	FuzzyEn + PSD	87.7 ± 4.17	0.84 ± 0.08	87.91 ± 5.12	4.42 ± 5.09
Our method	FuzzyEn + PSD + Brain networks	98.01 ± 2.60	0.96 ± 0.05	98.30 ± 2.19	3.83 ± 5.05

#### 3.3.2. Performance evaluation of seizure prediction based on the CHB-MIT dataset

Table 6 shows that our approach has also achieved a good prediction performance on the CHB-MIT scalp EEG signals. The results obtained an average accuracy, AUC, F-score and FPR of 95.93%, 0.92, 94.97%, and 0.0473/h, respectively. The prediction accuracy of most patients exceeds 99% such as chb01, chb05, chb06, chb10, chb15, chb18, chb20, and chb23. However, not all patients have good prediction accuracy such as chb07, chb08, and chb14, which have an accuracy of less than 90%. The reason may be that these patients had relatively few training samples (3−5 seizures). Among them, the chb07 with only three seizures had the lowest accuracy of 85.12%. And FPR may be another reason for this unsatisfactory classification effect of chb07 and chb08. For these two patients, the FPR shows that it is easy to misjudge the interictal states as preictal states. Overall, our proposed approach was validated for predicting epileptic seizures, indicating that the optimal spatiotemporal feature set is effective. It was also found that the prediction performance varies greatly between different patients.

**TABLE 6:** Performance evaluation of our proposed method on the CHB-MIT dataset.

Patients	ACC (%)	AUC	F-score (%)	FPR (%)
chb01	99.95 ± 0.11	0.99 ± 0.01	99.95 ± 0.11	0.05 ± 0.11
chb03	97.36 ± 1.41	0.96 ± 0.01	98.32 ± 1.36	0.15 ± 0.33
chb05	99.36 ± 0.48	0.99 ± 0.01	99.36 ± 0.48	0.39 ± 0.87
chb06	99.65 ± 0.61	0.99 ± 0.01	99.65 ± 0.60	0.28 ± 0.58
chb07	85.19 ± 14.50	0.80 ± 0.29	74.14 ± 12.51	22.22 ± 38.49
chb08	85.28 ± 20.01	0.71 ± 0.40	87.77 ± 13.51	19.50 ± 43.60
chb09	88.33 ± 21.51	0.77 ± 0.43	79.76 ± 38.67	1.18 ± 2.36
chb10	99.72 ± 0.38	0.99 ± 0.01	99.71 ± 0.38	0.56 ± 0.77
chb14	91.09 ± 19.35	0.82 ± 0.38	93.74 ± 13.00	17.82 ± 38.69
chb15	99.12 ± 0.49	0.98 ± 0.01	99.16 ± 0.78	0.94 ± 1.25
chb18	99.48 ± 0.86	0.98 ± 0.02	99.49 ± 0.84	0.90 ± 1.81
chb20	99.17 ± 0.69	0.98 ± 0.01	99.16 ± 0.71	0.22 ± 0.49
chb21	98.57 ± 1.15	0.98 ± 0.01	98.68 ± 1.01	1.78 ± 1.45
chb23	99.72 ± 0.28	0.99 ± 0.01	99.73 ± 0.27	0.22 ± 0.50
Average	95.93 ± 5.74	0.92 ± 0.10	94.97 ± 8.40	4.73 ± 8.25

Table 7 shows that the average accuracy, AUC, F-score and FPR of using temporal features are 86.4, 0.81, 84.49, and 7.01%, respectively. While using the spatial features related to the brain network, the average accuracy, AUC, F-score and FPR are 82.19, 0.77, 82.66, and 11.17%, respectively. These results are consistent with that on the Kaggle dataset, indicating that the optimal set generated by fusing spatiotemporal features can significantly improve the prediction performance of epileptic seizures.

**TABLE 7:** Comparing the proposed spatiotemporal features method for seizure prediction with only temporal or spatial features on the CHB-MIT dataset.

Features		ACC (%)	AUC	F-score (%)	FPR (%)
Temporal features	FuzzyEn + PSD	86.40 ± 4.78	0.81 ± 0.05	84.49 ± 5.78	7.01 ± 6.45
Spatial features	Brain networks	82.19 ± 4.79	0.77 ± 0.10	82.66 ± 4.93	11.17 ± 8.74
Our method	FuzzyEn + PSD + Brain networks	95.93 ± 5.74	0.92 ± 0.10	94.97 ± 8.40	4.73 ± 8.25

#### 3.3.3. Comparison with existing state-of-the-art methods

Table 8 provides the comparison results on seizure prediction performance between our method and other existing state-of-the-art methods using the same datasets (American epilepsy society-Kaggle iEEG dataset and the CHB-MIT scalp EEG dataset). Syed (Usman et al., 2021) performed a deep learning approach that extracts both the handcrafted and the automated features as the input to an ensemble classifier of SVM, CNN, and LSTM, resulting in an accuracy of 95.53 and 96.05% on Kaggle and CHB-MIT datasets, respectively. A semi-dilated convolutional network (SDCN) was proposed by Hussein et al. (2021) which EEG signals were converted into a mage-like format by continuous wavelet transforms. The results finally achieved the AUC of 0.928 on the Kaggle dataset and a high accuracy of 98.82% on the CHB-MIT dataset. Xu et al. (2020) developed an end-to-end deep learning method with a higher AUC of 0.981 and 0.988 on the Kaggle dataset and CHB-MIT dataset, respectively. Truong et al. (2018) proposed a convolutional neural network extracting time and frequency domain information by using short-time Fourier transform (STFT), which has only obtained an average sensitivity of 75% and FPR of 0.21/h on the Kaggle dataset and the average sensitivity of 81.4% on CHB-MIT dataset.

**TABLE 8:** The comparison results between the proposed method and other existing state-of-the-art techniques.

References	Features	Classifier	Dataset	ACC (%)	AUC	F-Score (%)	FPR (/h)
Usman et al., 2021	CNN, Statistical and spectral moments	Ensemble of SVM, CNN and LSTM	Kaggle	95.53	−	SPE:95.81 SEN:94.20	−
			CHI-MIT	96.05	−	SPE:96.28 SEN:95.65	−
Hussein et al., 2021	Continuous wavelet transform	SDCN	Kaggle	−	0.928	SPE: 85.6 SEN:88.45	−
			CHB-MIT	98.82	0.97	SPE:98.90 SEN:98.75	0.06
Xu et al., 2020	CNN	CNN	Kaggle	−	0.981	SEN:93.5	0.063
			CHB-MIT	−	0.988	SEN:98.8	0.074
Truong et al., 2018	Spectrogram	CNN	Kaggle	−	−	SEN:75	0.25
			CHB-MIT	−	−	SEN:81.4	0.06
This manuscript	FuzzyEn + PSD + Brain networks	SVM	Kaggle	98.01	0.96	98.3	0.038
			CHB-MIT	95.93	0.92	94.97	0.047

Compared to the other existing state-of-the-art methods, our proposed approach achieves better FPR on both of these two datasets, illustrating that the probability of our error warning in predicting seizures is the lowest. Meanwhile, our method also obtains better performance than other methods on the Kaggle database. For the CHB-MIT dataset, the accuracy of our model is slightly lower than that of Hussein et al. (2021) and Usman et al. (2021), but the complex features and three classifiers combination required in the method proposed by Usman has caused high complexity and the sensitivity; and Hussein’s paper only achieved the accuracy of 88.45% on iEEG dataset. Furthermore, both of these two methods are not provided the indicator FPR. In summary, compared with the existing advanced techniques, our method still has certain advantages in seizure prediction on both intracranial and scalp EEG signals.

## 4. Conclusion

In this paper, epileptic spatiotemporal information is deeply mined from comprehensive multiple dimensions of time-frequency, non-linearity and brain network. A novel prediction model is proposed by using spatiotemporal information with optimal features strategy for seizures early warning. The optimal spatiotemporal features set was formed by screening the high independence and rich information of the extracted features. This feature set has been input into SVM for training and recognition. On the Kaggle intracranial EEG dataset, this model achieved an average accuracy of 98.01%, AUC of 0.96, F-Score of 98.3% and FPR of 0.0383/h, respectively; and On the CHB-MIT scalp EEG dataset, the average accuracy, AUC, F score and FPR were 95.93%, 0.92, 94.97%, and 0.0473/h, respectively. An ablation study was performed to compare our model with two other methods using only temporal features or spatial features. The results show that our method achieves more effective performance. Compared to other existing state-of-the-art approaches on the same datasets, this present method has certain advantages in prediction performance. It is further confirmed that our spatiotemporal information can effectively identify the preictal states, which is helpful to the early warning of seizures for the clinical epileptic patients.

## Data availability statement

The original contributions presented in this study are included in the article/supplementary material, further inquiries can be directed to the corresponding authors.

## Author contributions

LZ and FY designed the work and wrote the original manuscript. JZW, SH, and ZH contributed to the analysis of data. YL, JW, JY, and ZL contributed to the review and editing. ZL mainly responsible for this project. All authors contributed to the article and approved the submitted version.

## Footnotes

1
https://physionet.org/content/chbmit/

2
https://www.kaggle.com/c/seizure-detection/data
